# Interfacial Zinc Phosphate is the Key to Controlling Biocompatibility of Metallic Zinc Implants

**DOI:** 10.1002/advs.201900112

**Published:** 2019-05-17

**Authors:** Yingchao Su, Hongtao Yang, Julia Gao, Yi‐Xian Qin, Yufeng Zheng, Donghui Zhu

**Affiliations:** ^1^ Department of Biomedical Engineering University of North Texas TX 76207 USA; ^2^ Department of Materials Science and Engineering Peking University Beijing 100871 China; ^3^ Department of Biomedical Engineering Stony Brook University NY 11794 USA; ^4^ International Research Organization for Advanced Science and Technology Kumamoto University 2‐39‐1 Kurokami, Chuo‐Ku Kumamoto 860‐8555 Japan

**Keywords:** biodegradable metals, surface coating, zinc alloys, zinc hydroxide, zinc oxide

## Abstract

Recently emerged metallic zinc (Zn) is a new generation of promising candidates for bioresorbable medical implants thanks to its essential physiological relevance, mechanical strength, and more matched degradation pace to that of tissue healing. Zn‐based metals exhibit excellent biocompatibility in various animal models. However, direct culture of cells on Zn metals yields surprisingly low viability, indicating high cytotoxicity of Zn. This contradicting phenomenon should result from the different degradation mechanisms between in vitro and in vivo. To solve this puzzle, the roles of all major players, i.e., zinc phosphate (ZnP), zinc oxide (ZnO), zinc hydroxide (Zn(OH)_2_), pH, and Zn^2+^, which are involved in the degradation process are examined. Data shows that ZnP, not ZnO or Zn(OH)_2_, significantly enhances its biocompatibility. The mild pH change during degradation also has no significant impact on cell viability. Collectively, ZnP appears to be the key to controlling the biocompatibility of Zn implants and could be applied as a novel surface coating to improve biocompatibility of different implants.

## 1 Introduction

Metallic implants are the dominating players in clinical applications with about 70% of Food and Drug Administration (FDA) approved medical implants that are metal‐based, and only 30% are polymer‐based or soft materials. For example, they are the major ones applied in clinical treatments and therapies for the coronary angioplasty and orthopedic surgery.^1^ Currently, most coronary stents, orthopedic scaffolds, bone plates and screws, as well as artificial knees and hips are based on traditional inert metals such as titanium alloys, stainless steels, cobalt–chrome alloys, etc. They have many advantages including good machinability for complex structures and high mechanical support and durability.[qv: 1a,2] However, serious side effects also exist including late thrombosis, chronic inflammation, and inhibition of bone growth in young patients that necessitates a second removal surgery.[qv: 1a,3]

The emergence of biodegradable metals provides a solution to overcome these drawbacks associated with nondegradable implants as a transformative game changer.[qv: 1a,3a] They can degrade gradually in vivo after fulfilling their designed therapeutic roles.[qv: 3a] Magnesium (Mg) and iron (Fe) so far were the focus in the past when considering biodegradable metals.[qv: 3a,4] Recently, zinc (Zn) has emerged as a new generation of biodegradable biomaterials thanks to its promising degradation rate and biocompatibility, especially for cardiovascular and orthopedic applications.[qv: 2a,5] Compared to Mg and Fe, Zn possesses a more matched degradation pace to that of tissue healing.[qv: 2a,6] Moreover, Zn ion plays significant roles in cell metabolic activity and functions,[qv: 5b,7] bone growth, and maintaining cardiac function.[qv: 7a,8] This is an add‐on beneficial feature of Zn implants that release Zn ion during the degradation process. In fact, evidence showed that Zn‐based medical implants exhibited good biocompatibility in various animal models, including abdominal aortas of rabbits and rats,[qv: 6a,9] and femurs of rats and mice.^10^

Nonetheless, the toxicity of degradation products is always a concern which warrants more systematic investigations both in vivo and in vitro. Strikingly, most in vitro testing of Zn materials showed substantial cytotoxicity.[qv: 10b,11] Direct culture of cells on Zn surface even yielded significantly lower viability, indicating the strong cytotoxicity of Zn.^11^ This contradicting phenomenon between in vitro and in vivo should result from the different degradation mechanisms, i.e., the degradation rate and products. The degradation rate is the key factor determining the Zn ion release profile and pH change in the local environment. The degradation products should be mainly composed of insoluble Zn‐based compounds, including zinc phosphate (ZnP), zinc oxide (ZnO), and zinc hydroxide (Zn(OH)_2_).[qv: 6a,12] Thus, the goal of this study is to examine the effects of all the major players during the degradation process, i.e., ZnP, ZnO, Zn(OH)_2_, pH change, and Zn^2+^, on the biocompatibility of Zn implants, respectively.

Therefore, to determine the key factor controlling biocompatibility of metallic zinc implants, we designed and carried out the following experiments. First, different degradation products spontaneously formed during the degradation process were analyzed and identified both in vitro and in vivo. Next, each identified degradation product was specifically prepared as a monotonous coating on Zn substrate and examined for various cytocompatibility testing, respectively. In addition, we also controlled the degradation rate of the implants with different coatings at the same level to rule out its influence on the amount of Zn ion released and pH change during degradation. Last, the released Zn ion and pH change were also monitored to determine their impact on cytocompatibility, respectively.

## 2 Results

### 2.1 In Vivo Degradation of Zn Implants

**Figure 1:**
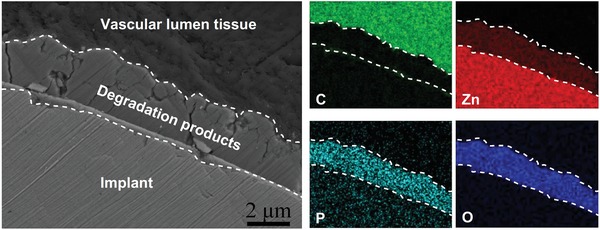
Zn stent in vivo degradation characterization in vascular tissue. Cross‐sectional morphology (backscattered electron images) and elemental mappings of Zn stent after 1 month of implantation in the rabbit abdominal aorta (C: carbon‐green; Zn: zinc‐red; P: phosphate‐turquoise; O: oxygen‐blue).

**Figure 2:**
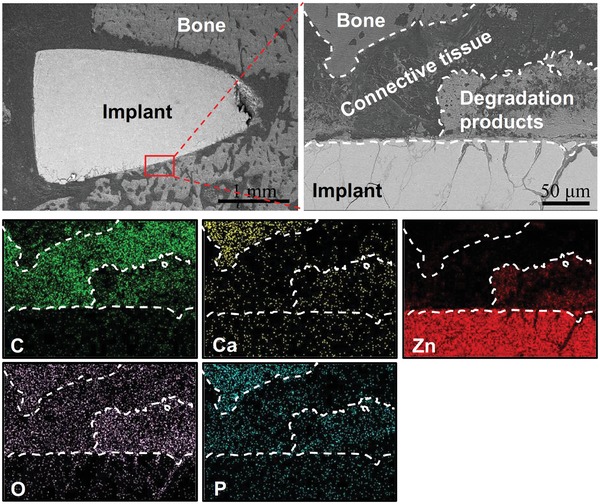
Zn bone pin in vivo degradation characterization in bone tissue. Cross‐sectional morphology (backscattered electron images) and elemental mappings of Zn after 1 month of implantation in the rat femur condyle (C: carbon‐green; Ca: calcium‐yellow; Zn: zinc‐red; O: oxygen‐purple; P: phosphate‐turquoise).

To analyze and identify the degradation products spontaneously formed during the in vivo degradation process, Zn stents and bone pins were prepared and implanted in the rabbit abdominal aorta and rat femur condyle, respectively. After 1 month of in vivo implantation, it could be observed that there was a dense and uniform degradation layer formed on the Zn stent surface, as shown in **Figure** **1**. The layer was mainly made of Zn, phosphorus (P), and oxygen (O) according to the elemental mappings. The vascular lumen tissue, i.e., endothelial monolayer, was smooth and uniform. There was no other connective tissue observed at the interface, indicating an excellent biocompatibility of the Zn stent. In contrast, there were some connective tissues formed at the bone tissue–implant interface after 1 month of in vivo implantation in the femur condyle (**Figure** **2**). The degradation products were mainly made of Zn, P, O, and calcium (Ca) and formed a segmental layer, not uniformly distributed on the Zn surface.

### 2.2 In Vitro Degradation of Zn Implants

**Figure 3:**
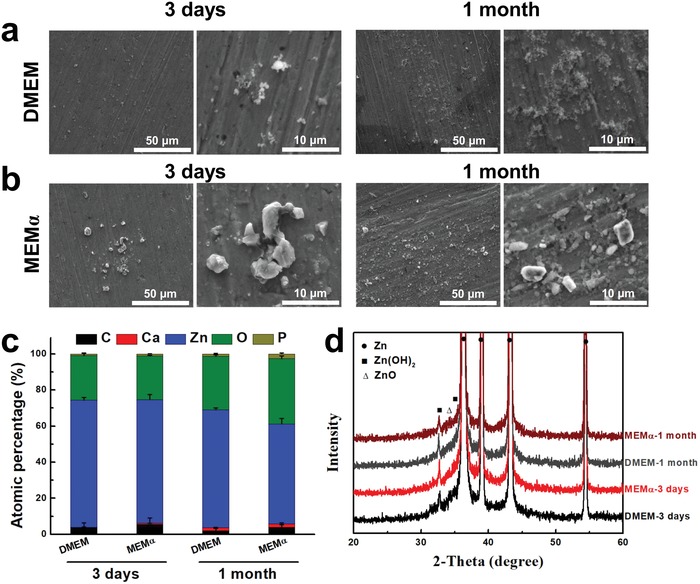
Zn degradation process in vitro. Surface morphology and composition of degradation products after 3 days and 1 month of immersion test in different cell culture media. a) Immersion test in DMEM, b) immersion test in MEMα, c) elemental composition by EDS, and d) phase composition by XRD (C: carbon; Ca: calcium; Zn: zinc; O: oxygen; P: phosphate).

In order to compare the in vivo and in vitro degradation mechanisms/products and their effects on biocompatibility, surface morphology and degradation products of Zn after 3 days and 1 month of immersion in different cell culture media were studied, as shown in **Figure** **3**. The surface kept flat with some white particles formed after 3 days in both cell culture media (Figure 3a). The white particles were mainly composed of Zn, P, O, and a small amount of Ca according to the energy‐disperse spectrometer (EDS) (Figure 3c). The degradation products were mainly Zn(OH)_2_ (wulfingite, PDF No. 38‐0385) and ZnO (zincite, PDF No. 36‐1451) indicated from the X‐ray diffraction (XRD) patterns (Figure 3d). The white particle‐like degradation products, mainly zinc phosphate (indicated by EDS), accumulated to form an incomplete thin layer on the Zn surface after 1 month of immersion in both the cell culture media, but the amount was too little to be detected by XRD.

### 2.3 Surface Coating Morphology and Composition

**Figure 4:**
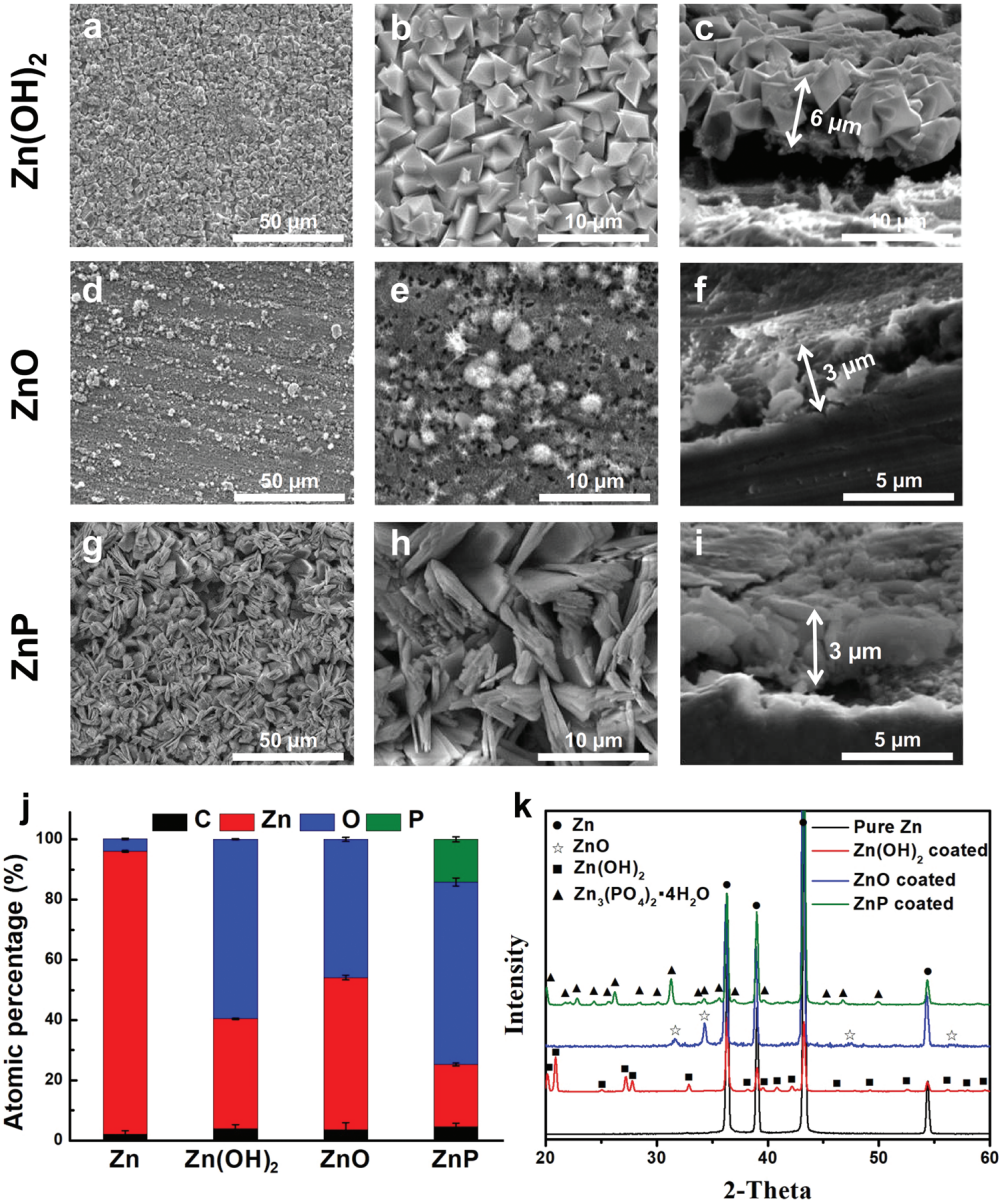
Surface and cross‐sectional coating morphology and phase composition of different Zn materials. a–c) Zn(OH)_2_ coating, d–f) ZnO coating, g–i) ZnP coating, j) elemental composition by EDS, and k) phase composition by XRD (C: carbon; Zn: zinc; O: oxygen; P: phosphate).

Next, to study the biocompatibility of each individual degradation product, every identified degradation product was specifically prepared as a monotonous coating on Zn substrate, as shown in **Figure** **4**. On the fresh‐grounded surface of Zn, there were some oxides formed according to the EDS results (Figure 4j), but too little to be detected by the XRD (Figure 4k). The three different coatings were shown in Figure 4a–i with different microsized cluster‐like morphologies, i.e., crystalloid particles for Zn(OH)_2_ coating, flat fluffy needles for ZnO coating, and thin flakes for Zn_3_(PO_4_)_2_·4H_2_O (ZnP, hopeite, PDF No. 37‐0465) coating. In order to keep similar degradation rates for all the Zn materials, coating thickness was optimized, i.e., about 6 µm for the Zn(OH)_2_ coating and 3 µm for the ZnO and ZnP coatings, respectively (Figure 4 c,f,i). The uniform coating structures and morphologies and the corresponding EDS and XRD data indicated the monotonous coating phase for each different coating.

### 2.4 Degradation Behavior of Zn Materials with Different Coatings

**Figure 5:**
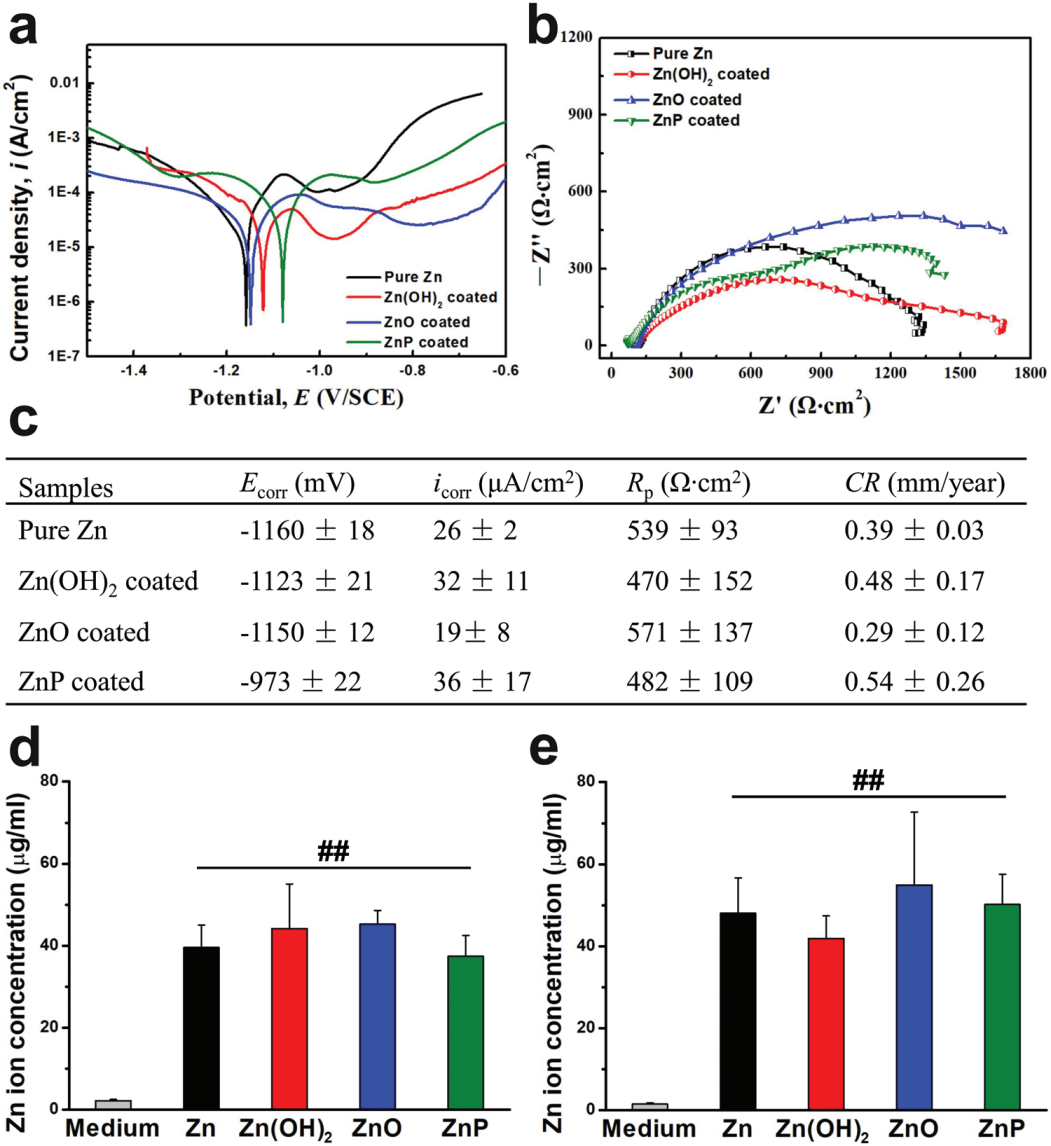
Electrochemical corrosion behaviors of different Zn materials. a) Potentiodynamic polarization, b) electrochemical impedance spectroscopy (EIS) in Hank's solution, c) electrochemical corrosion parameters of different samples, d) Zn ion concentration after 3 days of immersion in DMEM, and e) Zn ion concentration after 3 days of immersion in MEMα. ##*p*  <  0.005, compared with control group.

To rule out the effects of Zn^2+^ released and pH change on biocompatibility during degradation which was largely dependent on the degradation rate, we optimized each coating to result in a similar degradation performance in vitro. The electrochemical degradation behaviors of different coated Zn samples in Hanks' solution were shown in **Figure** **5**a–c. As compared to the uncoated sample, the corrosion potential (*E*_corr_) for all the coated samples showed slight shifts toward the positive direction, but the polarization current density (*i*_corr_), the polarization resistance (*R*_p_), and the calculated corrosion rate (CR) did not show significant differences for all the uncoated and coated samples (Figure 5c). The similar diameters of the semicircles for different samples indicated their similar degradation rate in Hanks' solution (Figure 5b). The Zn^2+^ concentrations after immersion in two cell culture media for 3 days were also monitored, as shown in Figure 5d,e. Zn^2+^ concentrations in both the control culture media were ≈2 µg mL^−1^, while Zn^2+^ released for the uncoated and coated samples in both cell culture media were ≈40 µg mL^−1^ after 3 days of immersion, indicating no significant differences for the Zn^2+^ released from different groups. Similar results were observed on pH change for all Zn materials, and there were slight increases in pH value after 3 days without significant differences among all the experimental groups (data not shown).

### 2.5 Cell Viability

**Figure 6:**
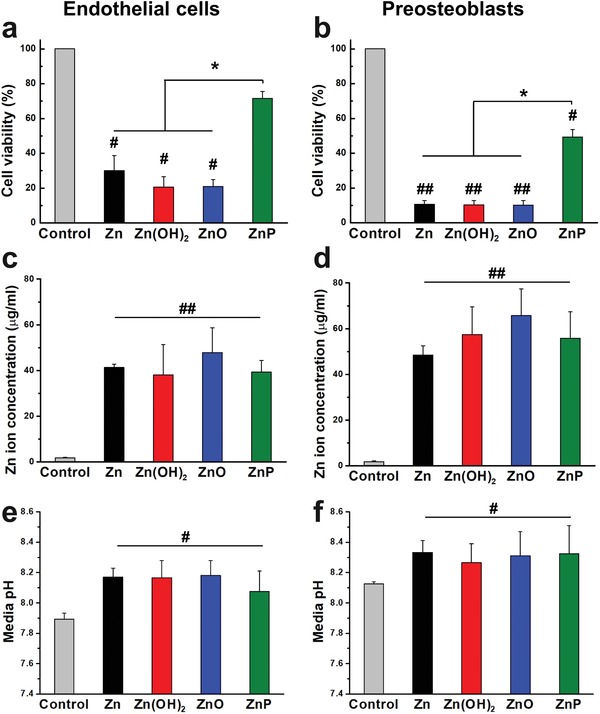
Cell viability by direct MTT assay. a) Endothelial cells and b) preosteoblasts, and the corresponding c,d) Zn ion concentration and e,f) pH change after 3 days of cell culture. #*p*  <  0.05, ##*p*  <  0.005, compared with control group; *p  <  0.05, compared between groups.

After ruling out the interferences of Zn^2+^ released and pH change during degradation on biocompatibility, next we determined the cytocompatibility of different coatings using direct MTT assay with endothelial cells and preosteoblasts, as shown in **Figure** **6**a,b, respectively. Cell viabilities of endothelial cells and preosteoblasts were 10–30% when cultured with Zn, Zn(OH)_2_, and ZnO‐coated samples, while the ZnP‐coated samples significantly improved cell viability of both cells when compared to the other experimental groups. The released Zn^2+^ and pH change were also monitored as shown in Figure 6c–f. The Zn^2+^ concentrations in the cell media after 3 days with uncoated and coated samples were ≈40 µg mL^−1^, significantly higher than the control group (Figure 6c,d), but no significant differences among all the experimental groups. This was consistent with the results from immersion testing (Figure 5d,e). In addition, all the experimental groups possessed similar and slight pH increments after 3 days of cell culture without significant differences (Figure 6e,f).

### 2.6 Cell Morphology

**Figure 7:**
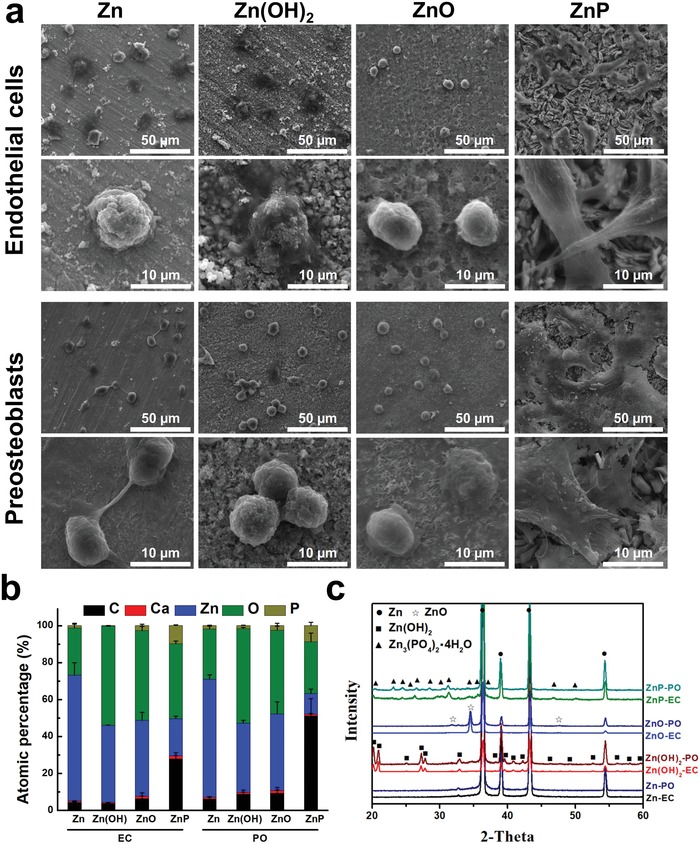
Cell adhesion morphology of endothelial cells and preosteoblasts on different Zn materials after 3 days. a) SEM images, b) elemental composition by EDS, and c) phase composition by XRD (C: carbon; Ca: calcium; Zn: zinc; O: oxygen; P: phosphate; EC: Endothelial cells; PO: Preosteoblasts).

Cell adhesion morphology of endothelial cells and preosteoblasts on different material surfaces were also examined after 3 days of cell culture (**Figure** **7**). Both cells showed round morphology and limited spreading behavior on the Zn surface, and neither Zn(OH)_2_ nor ZnO‐coated surfaces improved the cell adhesion and spreading (Figure 7a). In contrast, the ZnP coating significantly enhanced the cell adhesion and growth on its surface. Both cells showed high spreading morphology, and especially, the preosteoblasts interconnected to form a cell layer on the porous ZnP coating surface (Figure 7a). This was consistent with the EDS data (Figure 7b) that the C content increased significantly on the ZnP‐coated surface when compared with other groups. The XRD patterns of different coated samples after cell culture showed the same phase compositions for both cells when compared to the as‐prepared coatings (Figure 7c).

### 2.7 Hemocompatibility

**Figure 8:**
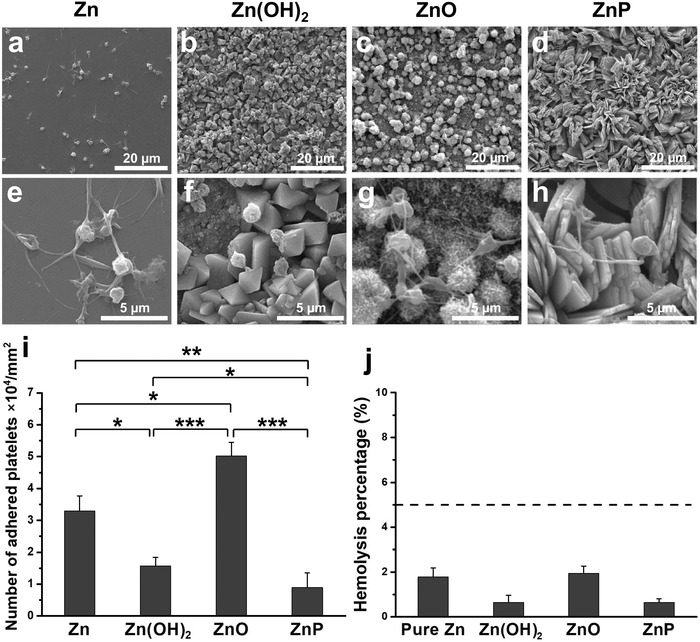
Hemocompatibility of different Zn materials. a–i) Platelets adhesion on experimental samples and j) hemolysis percentage, *p  <  0.05, **p  <  0.005, ***p  <  0.0005, compared between groups.

Moreover, platelet adhesion and hemolysis were also tested to verify the hemocompatibility of monotonous coatings on Zn surface, as shown in **Figure** **8**. The adhered platelets on the Zn‐ and ZnO‐coated surfaces had spreading pseudopodia and clumped together (Figure 8a,e,c,g), while those on the Zn(OH)_2_‐ and ZnP‐coated surfaces presented round morphology and limited spreading (Figure 8b,f,d,h). Although all these coatings had similar microscaled rough surfaces, there were significant differences for the adhered platelet number between different coated samples (Figure 8i). There were significantly more platelets on ZnO‐coated surface and significantly fewer platelets on Zn(OH)_2_ and ZnP‐coated surfaces when compared to uncoated Zn surface, with the least number of platelets on ZnP. The hemolysis rates for all experimental groups were far below 5% (Figure 8j), indicating the nonhemolytic properties of all materials.

## 3 Discussion

As one of the most promising biodegradable metals for cardiovascular and orthopedic applications, Zn has shown promising in vivo degradation rate and biocompatibility to potentially avoid the serious side effects associated with nondegradable implants. Our study also demonstrated the good biocompatibility of Zn in vivo as shown by the implant–tissue interactions at the interfaces (Figures 1 and 2). However, the direct cultures of both endothelial cells and preosteoblasts on Zn surface in the present study showed significantly lower viability in vitro (Figure 6a,b), consistent with previous reports,[qv: 10b,11] and indicating contradicting biocompatibility between in vitro and in vivo. This discrepancy should result from different degradation mechanisms during the short‐term in vitro cell culture model and long‐term in vivo implantation.

Zn materials show a moderate degradation rate between Fe and Mg materials,[qv: 2a,5b,6a,12] which is closer to the anticipated requirement for the biodegradable metallic implant materials. Metallic ion release and pH change in the local environment are largely determined by the degradation rate. There was little change on the pH value of the cell culture media with Zn materials during the cell culture period (Figure 6e,f). When compared with Mg materials, the pH change and hydrogen gas release for Zn materials are much less because of their different corrosion mechanism.[qv: 5c,6a,12,13] It has been previously indicated that Zn ion affects the cell responses in a concentration‐dependent manner in vitro.[qv: 7b,8,14] Although Zn ion plays significant and beneficial roles in cell behaviors and functions,[qv: 7b,8] overdosed Zn ion could induce harmful cellular responses in vascular smooth muscle cells and endothelial cells after its concentration reached a limit of 3.9 and 5.2 µg mL^−1^, respectively.^14^ In the present study, the Zn ion concentration reached 40 and 60 µg mL^−1^ after 3 days culture with endothelial cells and preosteoblasts, respectively (Figure 5d,e and Figure 6c,d), which are significantly higher than the concentration limits for normal cell behaviors and functions. We suspect that the Zn ion concentration in the local microenvironment around the Zn implant in vivo is at a similar level as in vitro although it is technically infeasible to measure it in real time. Most of the released Zn ion could accumulate in the culture media/tissue or rapidly form Zn‐based compounds as degradation products depending on the local microenvironment.

Theoretically, the main reactions and products of Zn degradation are as follows[qv: 6a,12]

(1) $$\mathrm{Zn}\to {\mathrm{Zn}}^{2+}+2e^{-}$$

(2) $$O_{2}+2H_{2}O+4e^{-}\to 4{\mathrm{OH}}^{-}$$

The Zn^2+^ could then quickly react with the OH^−^ and the HPO_4_^2−^ in surrounding physiological environment to form Zn(OH)_2_, ZnO, and ZnP[qv: 6a,12,15]

(3) $${\mathrm{Zn}}^{2+}+2{\mathrm{OH}}^{-}\to \mathrm{Zn}{\left(\mathrm{OH}\right)}_{2}$$

(4) $${\mathrm{Zn}}^{2+}+2{\mathrm{OH}}^{-}\to \mathrm{ZnO}+H_{2}O$$

(5) $$3{\mathrm{Zn}}^{2+}+2{\mathrm{HPO}}_{4}^{2-}+2{\mathrm{OH}}^{-}+2H_{2}O\to {\mathrm{Zn}}_{3}{\left({\mathrm{PO}}_{4}\right)}_{2}\cdot 4H_{2}O$$

**Figure 9:**
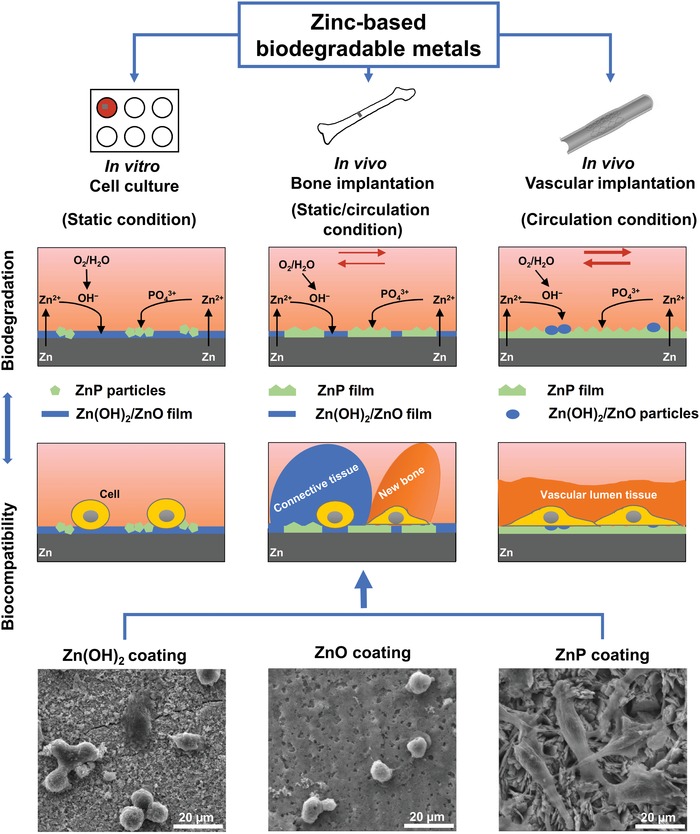
In vitro and in vivo degradation and biocompatibility mechanism of Zn‐based biodegradable metals.

The exact mechanisms and degradation products formation in vitro and in vivo could be different and must be determined by experiments. Thus, based on the experimental data in this study, we proposed the in vitro and in vivo degradation and biocompatibility mechanisms of Zn‐based biodegradable metals below as shown in **Figure** **9**.

The degradation behaviors of Zn materials are highly related with the microenvironments.[qv: 6a,10a] The oxygen partial pressure in different implantation environments has been shown to be one possible explanation in the previous studies on Zn‐ and Fe‐based biodegradable metals.[qv: 6a,10a,16] The static or circulation condition in the microenvironments could be another major parameter to affect the formation of degradation products. For in vitro scenario, Zn^2+^ is first released into media and the static condition facilitates the accumulation of the released OH^−^ at the interface followed by the formation of Zn(OH)_2_–ZnO layer through reactions as shown by Equations (3) and (4). A small amount of ZnP particles precipitate in this static condition (Figures 3 and 9). For in vivo scenario, when the Zn materials are implanted in vascular tissue, the blood circulation would greatly reduce the accumulation of OH^−^ at the interface, resulting in the formation of a uniform and dense layer of ZnP instead (Figures 1 and 9). However, when the Zn materials are implanted in bone tissue, the environment is a mixed static/circulation condition, leading to the formation of segmental layer of ZnP mosaicked with Zn(OH)_2_ and ZnO at the implant/bone interface (Figures 2 and 9), consistent with some earlier bone implantation studies.[qv: 10a,16a]

Among these three degradation products, i.e., ZnP, ZnO, and Zn(OH)_2_, ZnP is highly biocompatible while the other two are toxic as demonstrated by our data. ZnP‐coated Zn materials showed significantly improved cyto‐ and hemo‐compatibility when compared to the uncoated and Zn(OH)_2_–ZnO‐coated Zn materials (Figures 6, 7, 8, 9), consistent with a previous study that ZnP enhanced the cell adhesion of fibroblasts on the Ti surface.^17^ ZnO is one of the well‐known antibacterial agents and could be one possible explanation for the high cytotoxicity of Zn(OH)_2_–ZnO layer.^18^ The superior biocompatibility of ZnP is possibly due to its chemical structure, surface morphology, and stable solubility.^19^ The different biocompatibility of the spontaneously formed degradation products largely determines the different toxicity and tissue responses of the implants in vitro and in vivo as shown in Figure 9. The low biocompatible Zn(OH)_2_ and ZnO layers on the Zn surface lead to low cell viability and round cell morphology in the in vitro cell culture (Figures 6 and 7). The highly biocompatible and dense ZnP layer on the Zn stent surface promotes the cell adhesion and the vascular lumen tissue integration (Figure 1). The segmental ZnP layer on Zn bone implant may cause the formation of a fibrotic encapsulation (Figure 2). Compared to the direct tissue bonding of the Zn stent in the vascular lumen tissue, this connective tissue between the Zn implants and the bone tissue indicates a delayed osseointegration, but would be replaced with the newly formed bone eventually.[qv: 10a]

## 4 Conclusion

As novel promising bioresorbable medical implants, Zn‐based metals exhibited a contradicting biocompatibility in vitro and in vivo. Through comparing the Zn^2+^ release, pH change, and cytocompatibility of different degradation products, the different degradation mechanisms of Zn were proposed depending on various microenvironments. Data showed that ZnP, instead of ZnO or Zn(OH)_2_, significantly enhanced its biocompatibility at the similar levels of Zn^2+^ release and pH change. Especially, the dense and uniform ZnP layer spontaneously formed at the implant/tissue interface promoted the tissue integration, while the segmental interfacial layer of ZnP may cause a delayed tissue integration. Hence, the interfacial ZnP is the key controlling biocompatibility of metallic Zn implants. Moreover, ZnP could potentially be a promising coating material with stable chemical property to provide excellent biocompatibility for other biomedical implants.

## 5 Experimental Section

*In Vivo Degradation Analysis*: All experimental animal procedures were in accordance with institutional policies and the approval of the Animal Ethics Committee of the University. Two different animal models were used for in vivo implantation of Zn materials. For the cardiovascular stent application, rabbit abdominal aorta model was used. The surgical implantation was carried out following previous studies.[qv: 6a,20] Briefly, five adult Japanese rabbits with an average weight of 3–4 kg were used. Two zinc stents with dimensions of *Φ*3.0 × 10 mm were implanted in the abdominal aorta of each rabbit under angiography. For the orthopedic application, rat femur condyle model was used. The surgical implantation was carried out following previous studies.^10^ Briefly, five Zn cylinders were implanted in the drilled holes (*Φ*2.0 × 5 mm) through the distal of the epiphysis gap in the femoral lateral condyle of five male Sprague Dawley rats (3 months old, 180–220 g).

All animals were euthanized after 1 month of implantation. Tissues containing the implants were harvested and embedded in resin and then cut into cross‐sections of 1–1.5 mm thicknesses. After surface grinding and polishing, the cross‐sections were coated with gold and then observed by scanning electron microscopy (SEM, Hitachi S‐4800, Japan) equipped with an EDS to obtain backscattered electron images and elemental mapping.

*In Vitro Degradation Analysis*: The immersion degradation behaviors of Zn were tested for 3 days and 1 month in the cell culture media.[qv: 5a,10a,21] Briefly, Zn discs with dimensions of 10 mm × 4 mm (99.99%+ purity, Goodfellow, US) were incubated in two different cell culture media: Dulbecco's modified Eagle medium (DMEM, ATCC, US) and minimum essential media alpha (MEMα, Gibco, US) with 10% serum, respectively. The Zn ion concentration was monitored during the degradation process with a Zn colorimetric assay kit (Bio‐Vision, US). The surface morphologies after 3 days and 1 month of degradation were observed with SEM and EDS. The phase compositions of the degradation products were identified by XRD (Rigaku Dymax, Japan) with a scan rate of 4° min^−1^ using Cu Kα radiation at 40 kV and 44 mA.

*Coating Preparation and Characterizations*: Zn discs were grounded with sandpaper up to 1500 grit, and then ultrasonically cleaned in alcohol for 5 min. Chemical immersion coating methods with different coating solutions and temperatures were applied for the three coatings.^22^ A zinc‐containing alkali solution was prepared as follows: 10 mL of 1.0 m Zn(NO_3_)_2_ solution was added into 160 mL of 1.0 m KOH solution and stirred sufficiently, and the precipitate was then separated and removed after centrifugation. Zn samples were dipped in the coating solution for 10 min at room temperature and 80 °C to obtain the Zn(OH)_2_ coating and ZnO coating, respectively. A zinc‐containing acidic solution was prepared with the same volume of 0.15 m Ca(NO_3_)_2_ and 0.15 m H_3_PO_4_. The pH value of the solution was adjusted to 2.2 with 0.1 m NaOH solution. Zn samples were dipped in the coating solution for 5 min at room temperature to prepare the ZnP coating. After the coating preparation, all the coated samples were rinsed with deionized water and dried in air. Surface and cross‐sectional coating morphology and phase composition of different coatings were characterized using SEM, EDS, and XRD.

*Electrochemical Corrosion Behavior*: The electrochemical tests, including potentiodynamic polarization and electrochemical impedance spectroscopy (EIS) tests, were performed with an electrochemical station (Princeton Versa STAT3, USA) in Hanks' solution at 37 ± 0.5 °C.[qv: 21b,23] Briefly, an Ag/AgCl saturated KCl was used as the reference electrode, a platinum plate acted as the counter electrode, and the working electrode was different Zn disc samples with an exposed surface area of 0.2826 cm^2^. The EIS test was performed from 1 MHz down to 1 mHz with a potential amplitude of 10 mV. Afterward, the potentiodynamic polarization test was performed at a scanning rate of 1 mV s^−1^ and a potential range of ±0.5 V versus the open circuit potential. The values of polarization parameters were obtained by using CorrView software. The CR was calculated according to the following equation[qv: 22c,24]

(6) $$\mathrm{CR}=3.27\times 10^{-3}\cdot \frac{i_{\mathrm{corr}}}{\rho }EW=14.97\times 10^{-3}i_{\mathrm{corr}}$$

where *i*_corr_ is the corrosion current density (µA cm^−2^).

*Cell Viability and Morphology*: Human endothelial cells (EA.hy926, ATCC CRL‐2922, US) and murine calvarial preosteoblasts (MC3T3‐E1, ATCC CRL‐2593, US) were used to evaluate the cytocompatibility of uncoated and coated samples using the direct assay method.^25^ The cell culture procedure can be found in previous publications.^26^

Cells were seeded onto the sample surfaces in a 24‐well plate with a density of 5 × 10^4^ per well and cultured in the corresponding cell culture media for 3 days. Afterward, the media were extracted to measure the Zn ion concentration with a Zn colorimetric assay kit (Bio‐Vision, US). The cell viability was measured with the MTT assay (Thermo Fisher Scientific, US). To avoid the interaction of the Zn substrate with the tetrazolium dye in MTT kit, the cells on the sample surface were first detached using the trypsin treatment, and then cultured in 96‐well plates for up to 4 h for recovery. Afterward, the absorbance (*A*) of MTT assay was measured by a plate reader (Cytation 5, Biotek, US) at 562 nm. Culture media with cells was used as a control group.

The cell morphology was observed by SEM after being fixed and dehydration. Briefly, the samples with cells cultured for 3 days were fixed with 4% paraformaldehyde (PFA, Affymetrix, US) and 2% glutaraldehyde solution (Fisher Chemical, US) for 1 h at room temperature, followed by dehydration process with gradient alcohol solutions (30%, 50%, 70%, 90%, and 100%) and hexamethyldisilazane for 10 min, respectively. The samples were gold coated before SEM characterization.

*Hemocompatibility Evaluation*: The hemolysis and platelet adhesion tests were performed according to previous studies.[qv: 10a,26a] Briefly, 4 mL of healthy human blood (anticoagulant with 3.8% citric acid sodium, Zen‐Bio, US) was diluted with 5 mL of saline solution. All samples were precultured with 9.8 mL of saline solution for 30 min and 0.2 mL of diluted blood was added and incubated for 1 h. Also, 9.8 mL of deionized water and saline solution were also incubated with 0.2 mL of diluted blood as the positive and negative control, respectively. After centrifuging at 3000 rpm for 5 min, the supernatants were collected in 96‐well plates and the absorbance (*A*) was measured by a plate reader (Cytation 5, Biotek, US) at 545 nm. The hemolysis rate (HR) was calculated by the following equation: Hemolysis = (*A*_sample_ − *A*_negative_)/(*A*_positive_ − *A*_negative_).

Platelet‐rich plasma (PRP) with a platelet density of 10^8^ µL^−1^ (Zen‐Bio, US) was used for platelet adhesion test. 80 µL of PRP was overlaid on each sample surface and incubated at 37 °C for 1 h. The adherent platelet morphology was observed by SEM after fixation and dehydration in the same method with the cell morphology observation as described above. The adherent platelet number was counted by using Image J on five SEM images for each sample.

*Statistical Analysis*: Data were presented as mean ± standard deviation. Statistical analysis was performed with SPSS 18.0 software package (SPSS Inc. Chicago. USA) by one‐way analysis of variance (ANOVA) and post hoc Tukey's multiple comparison tests.

## Conflict of Interest

The authors declare no conflict of interest.
